# Plasma D‐dimer predicts poor outcome and mortality after spontaneous intracerebral hemorrhage

**DOI:** 10.1002/brb3.1946

**Published:** 2020-11-11

**Authors:** Qi Zhou, Daming Zhang, Xin Chen, Zhao Yang, Zhihui Liu, Baixing Wei, Mei Jin, Kairu Feng, Chunmei Guo, Junying Sun, Sheng Chen, Ruijia Zhang, Xiai Piao, Ilgiz Gareev, Zhenying Sun, Xiaoxiong Wang, Lili Li, Shiguang Zhao, Guang Yang

**Affiliations:** ^1^ Research Administration Office The First Affiliated Hospital of Harbin Medical University Harbin China; ^2^ Department of Neurosurgery The First Affiliated Hospital of Harbin Medical University Harbin China; ^3^ Institute of Brain Science Harbin Medical University Harbin China; ^4^ Department of Neurosurgery The Fourth Hospital of Harbin Medical University Harbin China; ^5^ Department of Neurosurgery The Second Affiliated Hospital of Harbin Medical University Harbin China; ^6^ Harbin Medical University Harbin China; ^7^ Bashkir State Medical University Ufa Russia

**Keywords:** D‐dimer, mortality, outcome, spontaneous intracerebral hemorrhage

## Abstract

**Background:**

The elevation of plasma D‐dimer levels may predict a higher risk of thrombosis and play a role in the pathological process of patients after spontaneous intracerebral hemorrhage (ICH). However, its function in predicting the prognosis of ICH has not been verified on large cases.

**Patients and Methods:**

Retrospective cohort study of 1,332 consecutive patients with spontaneous ICH at an academic medical center was conducted. Functional outcome at three months after ICH was dichotomized using the modified Rankin Scale (0–2 versus 3–6). D‐dimer level in blood was analyzed within 1 hr of admission. An ICH outcome score combining D‐dimer level for evaluating poor functional outcome and mortality was tested.

**Results:**

The proportion of patients with poor functional outcome and mortality at three months was significantly higher in patients with elevated D‐dimer level (*p* < .001). Multivariable analysis demonstrated that elevated D‐dimer level was an independent predictor of poor functional outcome (odds ratio 1.486, 95% confidence interval 1.086–2.060, *p* = .014) and mortality (odds ratio 2.015, 95% confidence interval 1.186–3.423, *p* = .01). An increasing ICH outcome score combining D‐dimer level was associated with increased poor functional outcome and mortality.

**Conclusions:**

Elevated plasma D‐dimer level after spontaneous ICH is associated with poor functional outcome and mortality. The study suggests that elevated D‐dimer level has a predictive value for outcome and mortality in patients with spontaneous ICH.

## Graphical abstract

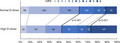

## 1 INTRODUCTION

Spontaneous intracerebral hemorrhage (ICH) is one of the leading causes of stroke‐related fatality and disability, which affects approximately 2 million people in the world each year (Cordonnier et al., 2018). In spite of advances in acute treatment strategies, prevention remains the most effective approach to reduce the significant impact of ICH (Hemphill et al., 2015).

There have been several reports assessing the risk factors for patients with ICH (Guo et al., 2017; Shoamanesh et al., 2018). Coagulation disorders and impaired hemostasis are established risk factors associated with ICH (Carpenter et al., 2016). Because endothelial injury often occurs after ICH which may lead to coagulation and fibrinolysis activation, D‐dimer will be released into the bloodstream as a fibrin degradation product. The elevation of plasma D‐dimer levels has been associated with a higher risk of thrombosis and an unfavorable neurologic outcome (Fukuda et al., 2018). Moreover, D‐dimer is also easily measurable in routine medical practice; therefore, the utility of D‐dimer as a blood biomarker assessing late clinical severity of ICH may add early prognostic information and be a suitable target for therapeutic research.

Recently, several studies reported that an elevated serum D‐dimer level could predict poor functional outcomes after ICH; however, there was limited data regarding the association between D‐dimer and outcome of ICH patients in any large study (Delgado et al., 2006; Hu et al., 2014). Therefore, the purpose of this study was to investigate whether D‐dimer level, detected within 1 hr of admission, can be an effective early indicator for poor functional outcome and mortality at three months in ICH patients based on large number of cases in a single center.

## 2 PATIENTS AND METHODS

### 2.1 Patient characteristics

Patients, aged ≥18 years, presenting directly to The First Affiliated Hospital of Harbin Medical University with spontaneous ICH between January 2016 and December 2016 were prospectively enrolled in an observational cohort study. Patients with ICH attributed to trauma, hemorrhagic conversion of ischemic stroke, structural lesion, or vascular malformation were excluded. Demographic and clinical data were systematically collected through interviews with patients and family members and through a retrospective review of hospital medical records. A non‐contrast brain CT scan was performed within 30 min of admission in patients with clinical suspicion of ICH. Hematoma volumes were measured on industry standard DICOM images using 3D Slicer 3.6.1 open source software (SPL, Harvard Medical School, Boston, USA) by a semi‐automated process. The Glasgow Coma Scale (GCS) score was prospectively recorded at the time of initial evaluation by a trained neurologist or neurosurgeon. Functional outcome was evaluated by modified Rankin Scale (mRS) after three months (poor functional outcome: mRS score, 3–6; favorable outcome: mRS score, 0–2). The study protocol was approved by the Institutional Review Board of the First Affiliated Hospital of Harbin Medical University.

### 2.2 D‐dimer measurement

Blood samples were obtained within 1 hr of admission and to analyze D‐dimer concentrations using standard techniques (CA‐7000 Sysmex; Dade Behring). D‐dimer was treated as a dichotomous variable. The value of D‐dimer ≥ 0.55 mg/L fibrinogen equivalent units (FEU) was defined as elevated.

### 2.3 Statistical analysis

Statistical analysis was performed using SPSS for Mac (version 21.0, IBM Corp.). Categorical variables were expressed as counts (percentage), whereas continuous variables were expressed as mean ± *SD* or median (interquartile range [IQR]) values. The differences between patients with and without high D‐dimer level on admission were examined using the χ^2^ test, the *t* test, or the Mann–Whitney *U* test as appropriate. Multiple logistic regression was calculated to identify predictors for poor functional outcome and mortality after incorporating the variables that revealed to be associated with mortality and poor outcome after three months in univariate analyses (*p* < .05). An ICH outcome score combining D‐dimer level was conducted by predictors which were screened using a multiple logistic regression analysis. The c‐statistic (area under the curve) was used to measure the goodness of fit. Statistical significance was defined as *p* < .05 for all tests.

## 3 RESULTS

**Figure 1:**
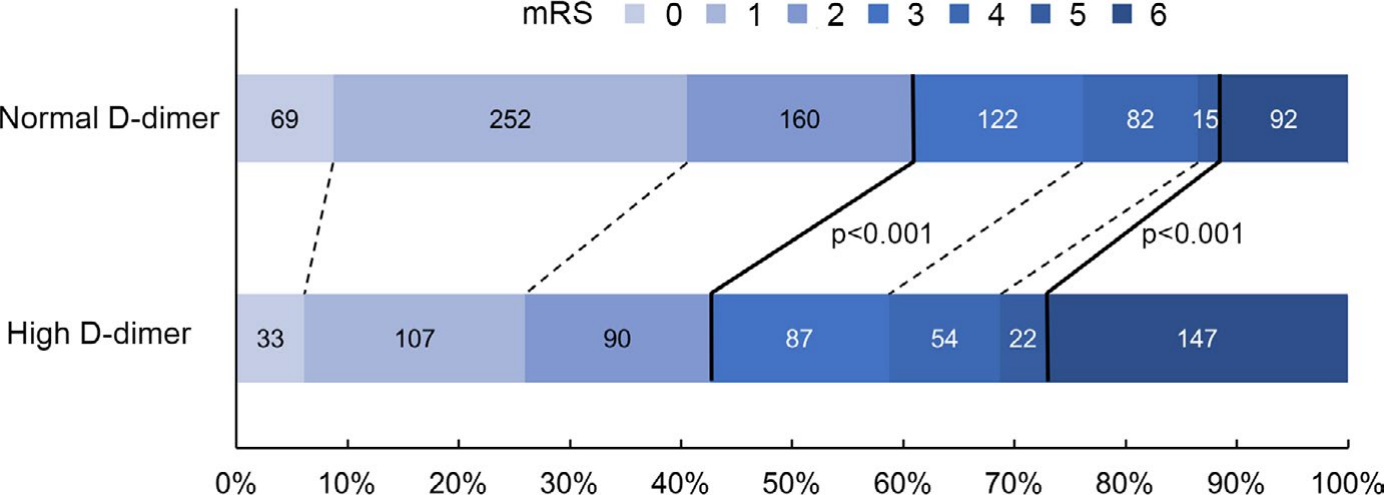
Distribution of modified Rankin Scale (mRS) according to serum D‐Dimer level. The bold line separates favorable (mRS, 0–2) and poor outcome (mRS, 3–6), or survival and death

We included 1,332 patients (mean 65 ± 14 years old, 52.1% female) with requisite data for analysis. D‐dimer was elevated in 540 of 1,332 patients with SICH (40.5%). Age, sex, heart rate, initial neurological status (NIHSS and GCS), hematoma location, midline shift, HCT, glucose, and fibrinogen were significantly associated with elevated plasma D‐dimer level. Patients with higher initial hematoma volume were more likely presented with D‐dimer elevation (*p* < .001). A more complete summary of the patients’ demographic and clinical characteristics is shown in table 1. The proportion of patients with poor functional outcome and mortality at three months was significantly higher in patients with elevated D‐dimer level (mRS at 3 months = 3–6: high 310/540 [57.4%] versus normal 311/792 [39.3%], *p* < .001; mortality at 3 months: high 147/540 [27.2%] versus normal 92/792 [11.6%], *p* < .001; Figure 1).

**Table 1:** Characteristics of ICH patients according to plasma D‐dimer levels on admission (*n* = 1,332)

Characteristics	Patients, No. (%)		*p* Value
	Normal D‐dimer (*n* = 792)	High D‐dimer (*n* = 540)	
Age, y	55.6 ± 10.3	62.8 ± 12.8	<0.001
Sex (male)	575 (72.6)	338 (62.6)	<0.001
History of hypertension	257 (32.4)	200 (37.1)	0.079
History of diabetes	92 (11.6)	80 (14.8)	0.085
First systolic BP, mm Hg	172.8 ± 29.8	174.9 ± 33.4	0.239
Heart rate	79.9 ± 16.2	84.3 ± 20.3	<0.001
Temperature, °C	36.6 ± 0.4	36.6 ± 0.6	0.175
Admit GCS	15 (12–15)	13 (7–15)	<0.001
Admit NIHSS	8 (3–16)	12 (6–20)	<0.001
Anticoagulant	104 (13.1)	84 (15.6)	0.207
Antiplatelet	126 (15.9)	103 (19.1)	0.149
Initial hematoma volume, ml	12.6 (4.9–26.7)	16.3 (6.3–45.1)	<0.001
IVH	190 (24.0)	178 (33.0)	<0.001
Location of ICH			
Lobar	79 (8.8)	67 (12.4)	0.032
Deep	435 (54.9)	278 (51.5)	
Infratentorial	56 (7.1)	52 (9.6)	
Midline shift, mm	2.6 (0–4.7)	2.9 (0–5.3)	0.043
Admission HCT, %	42.3 (39.6–45.2)	40.8 (37.3–44.1)	<0.001
Admission platelet count, K/uL	231.4 (193.9–269.0)	219.4 (184.6–267.2)	0.179
Glucose, mmol/l	6.51 (5.56–8.05)	7.35 (6.13–9.92)	<0.001
Admission INR	1.0 (1.0–1.1)	1.1 (1.0–1.1)	0.177
Admission PT, s	11.6 (11.2–12.1)	11.8 (11.3–12.3)	0.466
Admission APTT, s	24.8 (22.8–27.4)	24.7 (22.3–27.8)	0.648
Admission fibrinogen, g/l	2.44 (2.09–2.87)	2.80 (2.30–3.40)	<0.001
Surgery	181 (22.9)	130 (24.1)	0.605

Abbreviations: APTT, activated partial thromboplastin time; BP, blood pressure; GCS, Glasgow Coma Scale; HCT, hematocrit; ICH, intracerebral hemorrhage; INR, international normalized ratio; IVH, intraventricular hemorrhage on presentation; mRS, modified Rankin Scale; NIHSS, NIH Stroke Scale; PT, prothrombin time.

Values are *n* (%), mean ± *SD*, or median (interquartile range).

Table 2 shows the results of univariate logistic regression analysis of factors associated with poor functional outcome and mortality at three months. Univariate analysis demonstrated that elevated D‐dimer levels were significantly associated with poor functional outcome (OR 2.085 [95% CI: 1.669–2.604], *p* < .001) and mortality (OR 2.864 [95% CI: 2.149–3.817], *p* < .001). In multivariable logistic regression analysis, elevated D‐dimer level remained an independent predictor of poor functional outcome (OR 1.486 [95% CI: 1.086–2.060], *p* = .014) and mortality (OR 2.015 [95% CI: 1.186–3.423], *p* = .01), after adjustment for the other covariates that were marginally or significantly associated with poor functional outcome and mortality in univariate analysis and clinical characteristics (Table 3). To compare the impact of D‐dimer on the multivariable model, a sub‐analysis without D‐dimer was showed in Table S1.

**Table 2:** Univariate associations with poor functional outcome (mRS 3–6) and mortality

Variable	90‐day poor outcome			90‐day mortality		
	OR	95% CI	*p* Value	OR	95% CI	*p* value
Age, year	1.021	1.012–1.031	<0.001	1.012	1.000–1.023	0.043
Sex (male)	0.998	0.795–1.252	0.924	0.979	0.734–1.306	0.883
History of Hypertension	0.999	0.800–1.249	0.994	0.765	0.502–1.166	0.213
History of Diabetes	1.028	0.754–1.403	0.860	0.798	0.605–1.053	0.111
First systolic BP, mm Hg	1.009	1.006–1.013	<0.001	1.012	1.007–1.016	<0.001
Heart Rate	1.011	1.005–1.017	<0.001	1.025	1.018–1.032	<0.001
Temperature	1.479	1.162–1.883	0.001	2.015	1.552–2.615	<0.001
Admit GCS	0.797	0.771–0.825	<0.001	0.704	0.676–0.732	<0.001
Admit NIHSS	1.091	1.076–1.107	<0.001	1.132	1.112–1.152	<0.001
Anticoagulant	1.086	0.804–1.468	0.590	1.430	1.001–2.044	0.049
Antiplatelet	1.023	0.774–1.353	0.871	1.293	0.923–1.814	0.136
Initial hematoma volume, ml	1.020	1.000–1.000	<0.001	1.028	1.022–1.034	<0.001
IVH	1.379	1.062–1.789	0.016	2.118	1.470–3.051	<0.001
Location of ICH	1.177	0.917–1.511	0.201	1.372	0.961–1.958	0.081
Midline shift, >10 mm	2.061	1.167–3.641	0.013	3.883	2.140–7.046	<0.001
Admission HCT, %	1.000	0.979–1.022	0.979	0.985	0.957–1.013	0.293
Admission platelet count, K/uL	1.001	1.000–1.002	0.194	1.001	0.999–1.003	0.229
Glucose, mmol/L	1.114	1.073–1.156	<0.001	1.182	1.136–1.230	<0.001
Admission INR	1.788	1.004–3.184	0.048	2.931	1.611–5.330	<0.001
Admission PT, s	1.038	0.987–1.092	0.144	1.099	1.041–1.161	0.001
Admission APTT, s	0.996	0.974–1.017	0.694	1.000	0.972–1.028	0.978
Admission Fibrinogen, g/L	1.099	0.970–1.245	0.140	1.000	0.851–1.174	0.998
Admission D‐dimer, >0.55 mg/L FEU	2.085	1.669–2.604	<0.001	2.864	2.149–3.817	<0.001
Surgery	1.229	0.955–1.580	0.109	0.605	0.425–0.862	0.005

Abbreviations: APTT, activated partial thromboplastin time; BP, blood pressure; CI, confidence interval; GCS, Glasgow Coma Scale; HCT, hematocrit; ICH, intracerebral hemorrhage; INR, international normalized ratio; IVH, intraventricular hemorrhage on presentation; mRS, modified Rankin Scale; NIHSS, NIH Stroke Scale; PT, prothrombin time.

Values are *n* (%), mean ± *SD*, or median (interquartile range).

**Table 3:** Multiple associations with poor functional outcome (mRS 3–6) and mortality

Variable	90‐day poor outcome			90‐day mortality		
	Adjusted OR	95% CI	*p* Value	Adjusted OR	95% CI	*p* Value
Age, y	1.015	1.002–1.029	0.025	1.016	0.995–1.037	0.468
First systolic BP, mmHg	1.006	1.001–1.011	0.014	1.007	0.999–1.014	0.075
Admit GCS	0.918	0.858–0.981	0.012	0.799	0.730–0.874	<0.001
Initial hematoma volume, ml	1.011	1.004–1.018	0.003	1.011	1.002–1.019	0.011
Admission PT, s	1.027	0.971–1.087	0.347	1.111	1.042–1.186	0.001
IVH	0.916	0.673–1.248	0.580	1.167	0.718–1.897	0.533
D‐dimer, >0.55 mg/L FEU	1.475	1.068–2.037	0.018	2.015	1.186–3.423	0.010

Abbreviations: BP, blood pressure; CI, confidence interval; GCS, Glasgow Coma Scale; IVH = intraventricular hemorrhage on presentation; mRS, modified Rankin Scale; NIHSS, NIH Stroke Scale; PT, prothrombin time.

**Figure 2:**
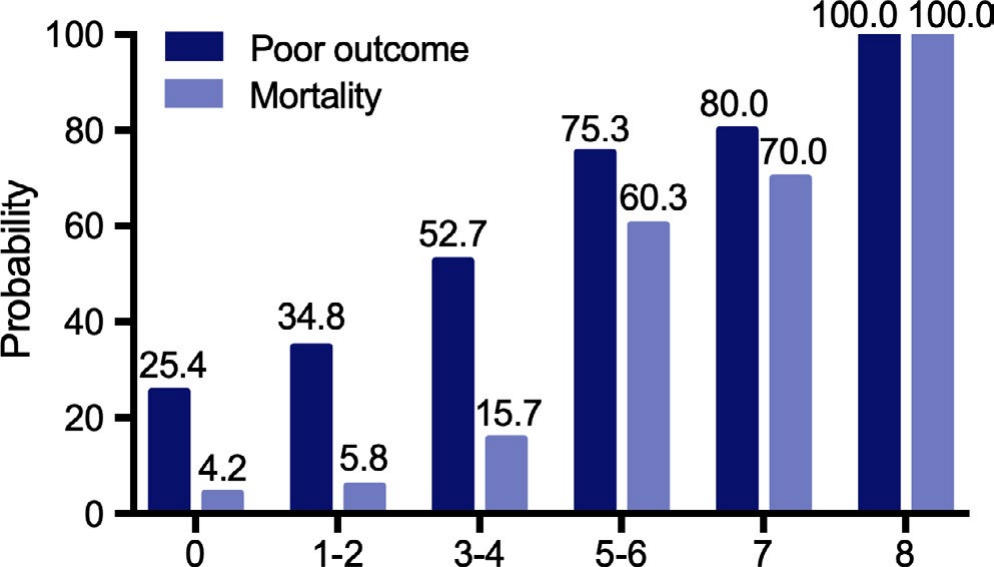
Probability of poor functional outcome and mortality according to ICH outcome score combining D‐dimer level category

Finally, to evaluate the impact of D‐dimer on the accuracy of poor functional outcome and mortality assessment, we designed an ICH outcome score combining D‐dimer level (ICH‐CDD). In multivariable logistic regression analysis, age, first systolic BP, admission GCS, initial hematoma volume, admission PT, intraventricular hemorrhage, and D‐dimer levels were also associated with poor functional outcome or mortality (Table 3). Each of these factors was incorporated into ICH‐CDD (total score ranging from 0–8 points; Table 4). The probability for poor functional outcome and mortality at three months for ICH‐CDD category is shown in Figure 2. A receiver operating characteristic (ROC) curve was created to assess the ability of ICH‐CDD to predict poorer functional outcome and mortality. ICH‐CDD showed an area under the curve (AUC) to predict functional outcome of 0.743 (95% CI: 0.692–0.795) and mortality of 0.826 (95% CI 0.782–0.871). In order to objectively evaluate ICH‐CDD, we assessed traditional ICH Score (Hemphill et al., 2001) and ICH Functional Outcome Score (ICH‐FOS; Ji et al., 2013) by ROC curve. The results showed the AUC of ICH Score was 0.655 (95% CI: 0.617–0.694) for functional outcome and 0.793 (95% CI: 0.742–0.844) for mortality; the AUC of ICH‐FOS was 0.698 (95% CI: 0.664–0.731) for functional outcome and 0.794 (95% CI: 0.746–0.842) for mortality. ICH‐CDD showed a potential advantage to predict poorer functional outcome and mortality relative to ICH Score and ICH‐FOS.

**Table 4:** ICH Outcome Score Combining D‐Dimer Level

Components	Points
Age	
≥80	1
<80	0
First systolic BP	
≥180 mmHg	1
<180 mmHg	0
Admit GCS	
3–4	2
5–12	1
13–15	0
Initial hematoma volume	
≥30	1
<30	0
Admission PT, s	
≥12.1 s	1
<12.1 s	0
IVH	
Yes	1
No	0
D‐dimer	
>0.55 mg/L FEU	1
≤0.55 mg/L FEU	0

Abbreviations: BP, blood pressure; GCS, Glasgow Coma Scale; IVH, intraventricular hemorrhage on presentation; PT, prothrombin time.

## 4 DISCUSSION

ICH has been studied from various standpoints with the aim of identifying the risk factor for life‐threatening complications and reliable prognostic criteria (James et al., 2017; Wilson et al., 2017). Some potential biomarkers have been observed in circulating blood of ICH patients. For example, high serum caspase‐3 and oxidized guanine species levels are associated with mortality (Lorente, Martín, González‐Rivero, et al., 2020; Lorente, Martín, Pérez‐Cejas, et al., 2020); however; low serum calcium levels are associated with hematoma expansion and worse outcome (Jafari et al., 2019). Our study demonstrated that higher plasma D‐dimer level, which is a fibrin degradation product, measured within 1 hr of admission, was significantly associated with poor functional outcome and increased mortality at three months in 1,332 patients with ICH, which is consistent with previous reports. More specifically, we further found that plasma D‐dimer levels were independent risk factors of mortality and poor outcome.

As we know, the elevated plasma D‐dimer levels may reflect a systemic hypercoagulability and cerebral microcirculation disorder (Di Castelnuovo et al., 2014). Cheng et al. investigated that plasma D‐dimer levels were significantly higher in ICH patients with deep venous thrombosis (Cheng et al., 2016). Similarly, recent studies showed that elevated D‐dimer increased incidence of thromboembolic events and systemic complications in subarachnoid hemorrhage (SAH) (Fukuda et al., 2017, 2018; Zheng et al., 2018). In our results, the elevation of D‐dimer showed a strong association with higher initial hematoma volume and intraventricular hemorrhage. To the best of our knowledge, our conclusion that D‐dimer is useful in predicting poor prognosis is based on the largest clinical sample data so far; however, the mechanism that elevated D‐dimer affects the prognoses of ICH patients still has not yet been established.

Although we have made significant progress in the management of ICH and monitoring patients throughout their hospitalization, the initial assessment of clinical prognosis using ICH score remains poor. Since D‐dimer levels have been demonstrated to be an independent predictor of poor outcome in ICH, they have not been previously incorporated into relevant prediction scores for functional outcomes and mortality of ICH patients (Chen et al., 2016; Hu et al., 2014). Considering D‐dimer's important ability to predict prognosis and because its levels are easily acquired in routine medical practice, we set a new ICH‐CDD for evaluating poor functional outcome and mortality in ICH patients. An increasing ICH outcome score combining D‐dimer level was associated with increased poor functional outcome and mortality. Although the AUC for poor functional outcome is not robust, ICH‐CDD could effectively evaluate and predict mortality at three months post event. Therefore, our data suggests that D‐dimer has an additive predictive value for outcome and mortality on conventional risk factors, but further studies still are needed to investigate whether this modified ICH score could replace or improve the contemporary prediction models.

We also acknowledge that our study has some limitations. First, although D‐dimer's role in coagulation and fibrinolytic systems is attractive, the pathophysiologic mechanism of D‐dimer in ICH has not been fully elucidated. Further studies need to reveal how D‐dimer levels affect hematoma enlargement. Second, our analysis was a retrospective design and conducted at a single institution. Further studies with a prospective and multicenter trial are needed to confirm our findings.

In conclusion, our study showed that elevated plasma D‐dimer level after spontaneous ICH is an independent risk factor for poor functional outcome and mortality. In addition, our findings also indicated that elevated D‐dimer level has an additive predictive value for outcome and mortality in patients with spontaneous ICH.

## CONFLICT OF INTEREST

The authors have no conflict of interest to declare.

## AUTHOR CONTRIBUTIONS

GY, QZ, DZ, and XC conceived and designed the study. ZY, ZL, BW, MJ, KF, CG, JS, SC, RZ, XP, IG, and LL acquired the data. QZ, DZ, XC, and XW analyzed and interpreted the data. GY, QZ, and XC drafted the manuscript. GY, QZ, DZ, XC, and ZS critically revised the article. GY and SZ approved the final version of the manuscript on behalf of all authors. QZ, DZ, and ZS statistically analyzed the data. GY and SZ supervised the study.

### Peer Review

The peer review history for this article is available at https://publons.com/publon/10.1002/brb3.1946.

## Supporting information

Table S1

## Data Availability

The data that support the findings of this study are available from the corresponding author upon reasonable request.
