# Supplementary material for: Plasma D‐dimer predicts poor outcome and mortality after spontaneous intracerebral hemorrhage
Source: Brain Behav. 2020 Nov 11;11(1):e01946. doi: 10.1002/brb3.1946 (PMC7821563; doi:10.1002/brb3.1946)
Supplement: Supplementary file 1 — Table S1 [file BRB3-11-e01946-s001.docx]

**Supplementary Table.** Multiple Associations with Poor Functional Outcome without D-dimer (mRS 3-6).

| **Variable** | **90 day poor outcome** | | |  | **90 day mortality** | | |
| --- | --- | --- | --- | --- | --- | --- | --- |
|  | **Adjusted OR** | **95%-CI** | **P Value** |  | **Adjusted OR** | **95%-CI** | **P Value** |
| Age, y | 1.020 | 1.007-1.033 | 0.002 |  | 1.021 | 1.001-1.041 | 0.036 |
| First systolic BP, mm Hg | 1.006 | 1.001-1.011 | 0.014 |  | 1.007 | 1.000-1.014 | 0.063 |
| Admit GCS | 0.916 | 0.857-0.980 | 0.010 |  | 0.800 | 0.731-0.874 | <0.001 |
| Initial hematoma volume, ml | 1.012 | 1.005-1.019 | 0.001 |  | 1.013 | 1.005-1.022 | 0.002 |
| Admission PT, s | 1.023 | 0.967-1.083 | 0.427 |  | 1.097 | 1.042-1.186 | 0.005 |
| IVH | 0.914 | 0.672-1.243 | 0.566 |  | 1.078 | 0.667-1.743 | 0.758 |

Abbreviations: BP = blood pressure; CI = confidence interval; GCS = Glasgow Coma Scale; mRS = modified Rankin Scale; NIHSS = NIH Stroke Scale; PT = prothrombin time.
